# Ascorbic Acid and BSA Protein in Solution and Films: Interaction and Surface Morphological Structure

**DOI:** 10.1155/2013/461365

**Published:** 2013-07-25

**Authors:** Rafael R. G. Maciel, Adriele A. de Almeida, Odin G. C. Godinho, Filipe D. S. Gorza, Graciela C. Pedro, Tarquin F. Trescher, Josmary R. Silva, Nara C. de Souza

**Affiliations:** Grupo de Materiais Nanoestruturados, Campus Universitário do Araguaia, Universidade Federal de Mato Grosso, 78600-000 Barra do Garças, MT, Brazil

## Abstract

This paper reports on the study of the interactions between ascorbic acid (AA) and bovine serum albumin (BSA) in aqueous solution as well as in films (BSA/AA films) prepared by the layer-by-layer technique. Regarding to solution studies, a hyperchromism (in the range of ultraviolet) was found as a function of AA concentration, which suggested the formation of aggregates from AA and BSA. Binding constant, *K*, determined for aggregates from BSA and AA was found to be about 10^2^ M^−1^, which indicated low affinity of AA with BSA. For the BSA/AA films, it was also noted that the AA adsorption process and surface morphological structures depended on AA concentration. By changing the contact time between the AA and BSA, a hypochromism was revealed, which was associated to decrease of accessibility of solvent to tryptophan due to formation of aggregates. Furthermore, different morphological structures of aggregates were observed, which were attributed to the diffusion-limited aggregation. Since most of studies of interactions of drugs and proteins are performed in solution, the analysis of these processes by using films can be very valuable because this kind of system is able to employ several techniques of investigation in solid state.

## 1. Introduction

Interactions between drugs and proteins have important implications for processes related to health [1]. The interactions can result in the formation of stable complexes (aggregates) that can have significant effects on the distribution, free concentration, and biological activity.

 In recent years, studies on the mechanisms of interactions between drugs and proteins have been performed with techniques such as spectroscopy, chromatography, and electrochemical and atomic force microscopy. Several factors can affect these mechanisms, such as concentration, temperature, and pH of solution. Self-assembly layer-by-layer deposition technique (LbL technique) is a convenient method for the formation of films from electrolytes. Through the change of experimental conditions, it is possible to control the film thickness at nanoscale and also manipulate the sequence of layers tuning them according to the wished property. Several experimental investigations have been performed using LbL technique, in which proteins, enzymes, nucleic acids, or carbohydrates are used [2–4]. LbL technique is based on adsorption process which can be understood by analyzing the adsorption kinetics (study of layer growth over time) and adsorption isotherms (study of adsorbed amount versus concentration). In addition, morphological analysis may also reveal information about the interaction between molecules [5].

 In this paper, we have investigated the behavior of the interaction of the ascorbic acid (AA) with bovine serum albumin (BSA) protein in the form of aqueous solution and BSA/AA films prepared by the LbL technique. Ascorbic acid can act as an antioxidant catalyst for tissue formation and wound healing, and inhibitor of tumor cell growth. These applications are possible due to interactions of ascorbic acid and human body protein. AA can bind to biological proteins modulating their activities. This bind process is determined by the behavior of interactions between drugs with proteins. In this context, albumin stands out for its ability to bind and transport small molecules [1, 6–9]. To the best of our knowledge there have been no studies on interaction between AA and proteins in LbL films.

## 2. Materials and Methods

 Ascorbic acid was purchased from Amresco. Bovine serum albumin, BSA (fraction V, purity 96–100%), was obtained from Acros Organics and used as received. The concentration of the dipping solutions (BSA and AA) for the preparation of BSA/AA films was set at 0.5 g/L. For the solutions of BSA, the pH was adjusted to 7 by adding NH_4_OH and to 3 for the solutions of AA by using HCl. The spectra of AA and BSA control solution are shown in the Supplementary Materials available online at http://dx.doi.org/10.1155/2013/461365. Both BSA and AA were used at concentrations less than 0.5 mg/mL, which were obtained by diluting the initial solution with aqueous 1 M HCl or 1 M NH_4_OH. BSA/AA films were adsorbed on quartz slides (36,0 mm × 14,0 mm × 1,0 mm). The fabrication procedures of layer by layer (LbL) followed essentially those described by Cardoso et al. [10]. The adsorbed amount, which is proportional to absorbance, was monitored by measuring the UV-vis absorption spectra with a double-beam Thermo Scientific spectrophotometer model Genesys 10. Surface morphological structure was investigated by using an LCD digital microscope (model 44340, Celestron, USA) and an atomic force microscope—AFM (model EasyScan II, Nanosurf Instruments, Switzerland) using the taping mode (256 × 256 pixels), under ambient conditions. *Gwyddion* software was used to determine the quantity of the surface forming structures. *ImageJ *software [11] was employed to determine the fractal dimension.

## 3. Results and Discussion

### 3.1. Study of Solutions

#### 3.1.1. Influence of Ascorbic Acid Concentration

**Figure 1:**
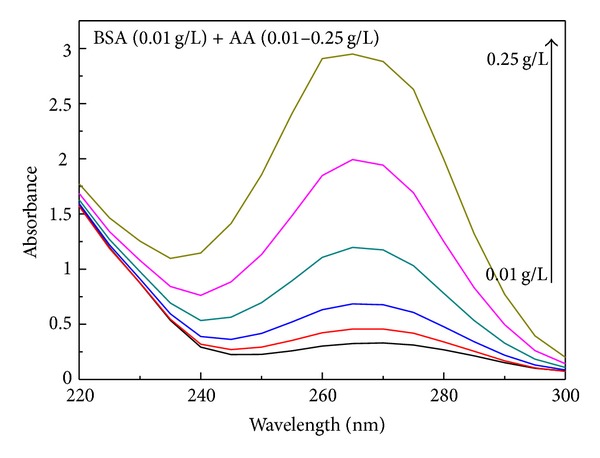
Spectra of BSA solution after addition of AA at different concentrations.

In order to study the interaction between AA and BSA, we have used UV-vis spectroscopy. This technique is a simple and effective method to investigate the molecular interaction and complex formation [12]. UV-vis analyses were performed for BSA in aqueous solution (pH 7, *c* = 0.01 g/L) and modified solution after the addition of AA (pH 3) at different concentrations. All of these experiments were carried out using 2.5 mL of BSA aqueous solution contained in a quartz cuvette. The amount of AA added was the same (40 *μ*L) for all concentrations examined (0.01, 0.03, 0.06, 0.12, 0.25, 0.37, and 0.5 g/L).  Figure 1 shows the UV-vis spectra at different concentrations for BSA solution with aliquot of 40 *μ*L of AA at pH 3. This experiment was repeated for solution without BSA at pH adjusted to 7 (Supplementary Materials) in order to rule out the effect of AA. We have observed a hyperchromism with increasing AA concentration in BSA. This effect can be associated to interaction of BSA with AA [4, 13, 14] and may be indicative of an increase in exposure of tryptophan to the solvent [15] due to a conformational change in the protein [12].

#### 3.1.2. Determination of Binding Constant

**Figure 2:**
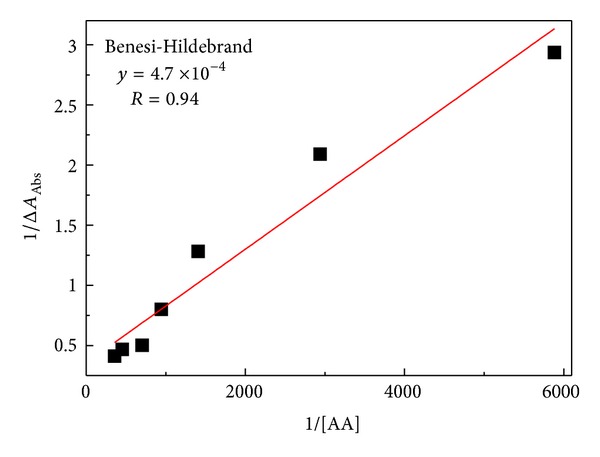
Linear regression for the reaction of BSA (0.01 g/L) with AA (0.01 to 0.5 g/L).

The binding constant, *K*, of AA with BSA was determined from the values of angular and linear coefficients, as shown in Figure 2 [14]. *K* value was found to be about 7.7 × 10^2^ M^−1^ obtained by using Benesi-Hildebrand equation:

(1) $$\begin{array}{c} \frac{1}{\Delta_{\text{Abs}}}=\frac{1}{\left[\text{BSA}\right]\left[\text{AA}\right]\varepsilon K}+\frac{1}{\left[\text{BSA}\right]\varepsilon }, \end{array}$$

where [BSA] and [AA] are concentration values in mol/L, *ε* is the absorption coefficient, and Δ_Abs_ is the change in absorbance at 280 nm for BSA bound and free.

The binding constant calculated is similar to that found for antineoplastic cisplatin (10^2^ M^−1^) and far from those found for other drugs, such as azidothymidine (AZT) (10^6^ M^−1^) and aspirin (10^4^ M^−1^). In addition, the bonding constant found here is lower than that determined for AA (10^4^ M^−1^) in lower concentrations examined [6, 16]. This suggests a weak interaction between AA and BSA, which would lead to a high free concentration of AA in blood.

#### 3.1.3. Influence of Contact Time

**Figure 3:**
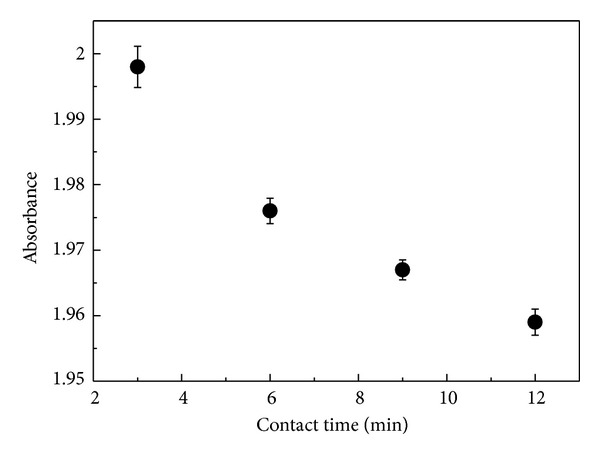
Absorbance as a function of contact time for the solution of BSA and AA.

Contact time between solutions is that measured from the moment that they are brought together. The influence of the contact time between AA and BSA on the aggregation process was investigated by using UV-vis spectroscopy, as shown in Figure 3. It is noted that the absorbance decreases with increasing the contact time. This hypochromism may be associated with protein folding and formation of aggregates. During this process, the exposition of tryptophans to solvent would decrease with increasing aggregate sizes leading to a decrease in absorbance. The process of diffusion-limited aggregation can play an important role in the formation of the fractal structures observed in this work [5].

### 3.2. Study of films

#### 3.2.1. Influence of Concentration

 In solid state, interactions between molecules can exhibit a different behavior from those found in a liquid state. Therefore, the investigation of properties of solid state films, such as surface morphology, can provide insights about these interactions. Here, we have studied the effect of the AA concentration on adsorption process (which is determined by the molecular interactions) of AA onto a single layer of BSA (BSA/AA film).

**Figure 4:**
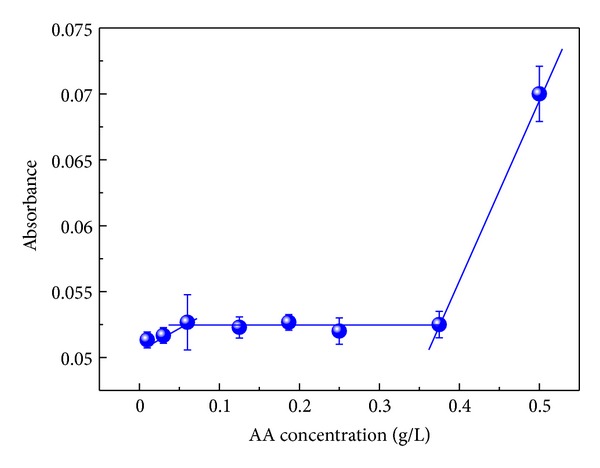
Absorbance versus concentration of AA. The immersion time into solutions of BSA and AA was 10 min. Solid lines are a guide to the eyes.

Figure 4 shows the behavior of an AA layer onto a single layer of BSA. The immersion time used for the solutions of BSA and AA was 10 min. As shown in Figure 4, there are two approximately linear regimes separated by a long plateau in the range of concentrations studied. For the first regime, it is noted that the adsorbed amount of AA increases with increasing concentration. This can be explained considering that as AA concentration increases, there are more molecules near surface of BSA film able to adsorb, and then as the substrate is immersed in the AA solution, the adsorbed amount increases. For the second regime, the linearity indicates that the number of sites for adsorption remains constant in this concentration range [17]. Finally, the third regime, which is an increase again, suggests that a second layer is being formed.

**Figure 5:**
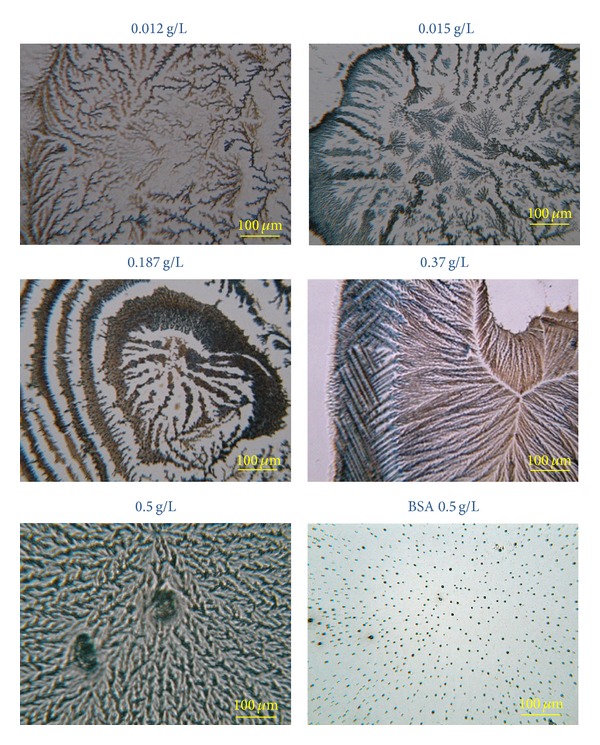
One-bilayer BSA/AA film with AA in top layer. AA concentration varied as indicated in the figure. The scale has length of 100 *μ*m. The last image corresponds to a film with a single layer of BSA.

Figure 5 shows an image sequence for a film in which AA was adsorbed onto BSA forming a top layer (BSA/AA film). It is observed that the presence of AA leads to the formation of fractal-shaped aggregates, which have their forms dependent on the AA concentration value. In the case of pure BSA films (0.5 g/L), small aggregates are observed but without fractal structures. It should be noted that although AA films present low coverage ratio of the layer of BSA, the images are reproducible. Fractal structures can be characterized by their fractal dimension. The fractal dimensions were determined using *ImageJ *software, which uses the box-counting method of fractal analysis. The values of fractal dimension, *D*_*f*_, found for each AA concentration employed were 1.69 (0.012 g/L), 1.71 (0.015 g/L), 1.75 (0.187 g/L), 1.87 (0.370 g/L), and 1.84 (0.5 g/L). It was shown that an increase occurs in the fractal dimension with the increase in the AA concentration.

 At low concentrations, the aggregates are organized as discrete structures on the surface, whereas for higher concentrations the aggregates are organized as compacted structures. Spontaneous organizations leading to fractal structures are common in natural systems and the understanding of bonding mechanisms of small particles to form large aggregates is interesting in the analysis and control of biomolecular interaction processes [18]. 

#### 3.2.2. Influence of Contact Time

The influence of the contact time between AA and BSA on the surface morphology of the BSA/AA films was investigated. The same volumes of solutions of BSA (0.5 g/L) and AA (0.5 g/L) were brought together each otherat different contact times. The LbL films were obtained by immersing the quartz slides by 3 min in the BSA + AA mixture. 

When the solution of AA is introduced in the same ratio (*v* : *v*) in solution of BSA for different contact times, the hypochromism effect is observed both on film and in solution. This phenomenon may be associated with the formation of aggregates which decreases the accessibility of the tryptophan, thereby reducing the intensity of absorbance.

**Figure 6:**
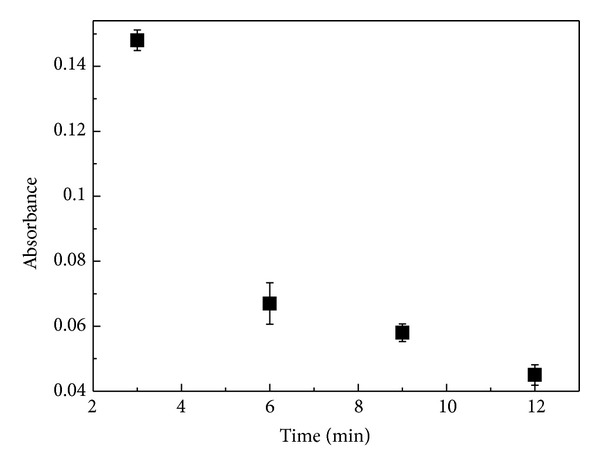
Absorbance as a function of contact time for BSA/AA films.

**Figure 7:**
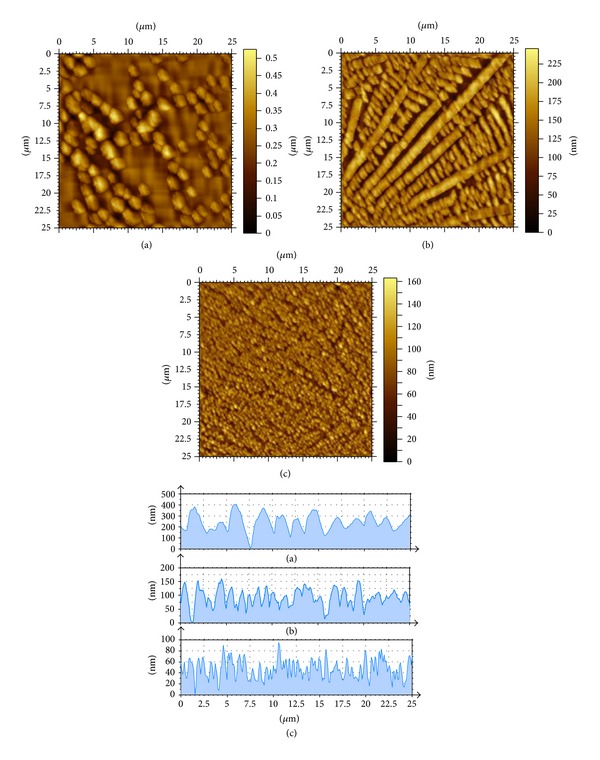
Images obtained by atomic force microscopy and corresponding profiles of self-assembled monolayers in time for solutions of BSA + AA obtained after (a) 3 min, (b) 6 min, and (c) 12 min of contact time.

In order to gain an insight about the hypochromism showed in Figure 6, an analysis of surface morphological structure of LbL BSA/AA films was performed.  Figure 7 shows images and the corresponding height profiles obtained by atomic force microscopy in scanning window of 25 *μ*m × 25 *μ*m for BSA/AA films.

Fractal-shaped aggregates that corroborate the hypochromism (Figure 6) are noted. These structures are also consistent with those observed in the images obtained by optical microscopy (Figure 5).

**Figure 8:**
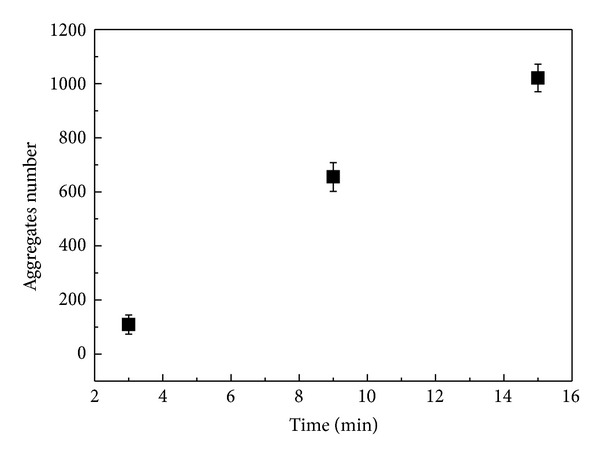
Number of aggregates as a function of contact time between BSA and AA for BSA/AA films.

Figure 8 displays the number of aggregates as a function of contact time for the morphological structures shown in Figure 7. It was observed that the number of aggregates increases with increasing the contact time. For short contact times, the interaction of BSA may be more intense with the solvent, which could explain the lower number of aggregates. Increasing the contact time, the interaction between BSA and AA should increase favoring an increase in the number of aggregates. The longer the contact time the more stable aggregates structures. Since the number of aggregates formed in solution depends on contact time, it is expected that the films present different morphological structures as this parameter is changed. Furthermore, it is well known that the aggregation in solution depends on experimental factors such as pH, concentration, temperatures or time [9, 19].

 The increase of number of aggregates as a function of the contact time, shown by the results of morphological analysis, corroborates the hypothesis that the aggregates make the tryptophan less accessible and this way the absorbance as a function of contact time decreases.

## 4. Conclusion

 We have investigated the interaction of AA with BSA in aqueous solution and also their effects on films by changing the concentration and contact time. In solution, hyperchromism indicated the formation of aggregates of AA and BSA. In addition, the binding constant of AA with BSA was found to be lower than those found for other drugs or even for lower concentrations of AA. This indicates a high free fraction of drugs. From the pharmacological viewpoint, only the free fraction can be transported by blood and other fluids to all tissues of the body. The fraction of drug bound to plasma protein forms a reversible complex, capable of dissociation. As the free part is used by the body, the linked part begins to dissociate. The increase in the free concentration of the drug increases its effect but also accelerates its elimination. For BSA/AA films, different regimes of adsorption (by using UV-vis spectroscopy) and surface morphology structures (by using optical microscopy) as a function of concentration were found. Furthermore, hypochromism as a function of the contact time was found, which was attributed to a decrease of accessibility in tryptophan to solvent due to aggregation. Atomic force microscopy for BSA/AA films revealed that the surface morphological structure of the films also depends on contact time; that is, for different contact times, different number and forms of aggregates were observed. In conclusion, films prepared by the layer-by-layer technique are interesting for drug-protein interaction studies because they exhibit structural organization and keep the active sites in the molecules immobilized. The use of this kind of film could pave the way for new investigations on the interactions of proteins with drugs, which usually employ pharmacokinetics techniques with solution samples.

## Supplementary Material

Figure A shows the AA and BSA solutions. The spectra at different concentrations for water (pH adjusted to 7) with aliquot of 40 *μ*L of AA at pH 3 are showed in Figure B. This experiment has been performed in order to rule out the effect of AA. The difference between the spectra of BSA (Figure 1 in the manuscript) and water (Figure B) reveals the behavior of BSA regarding to AA (Figure C).
