# Supplementary material for: Ascorbic Acid and BSA Protein in Solution and Films: Interaction and Surface Morphological Structure
Source: Biomed Res Int. 2013 Jul 25;2013:461365. doi: 10.1155/2013/461365 (PMC3741937; doi:10.1155/2013/461365)
Supplement: Supplementary file 1 — Figure A shows the AA and BSA solutions. The spectra at different concentrations for water (pH adjusted to 7) with aliquot of 40 μL of AA at pH 3 are showed in Figure B. This experiment has been performed in order to rule out the effect of AA. The difference between the spectra of BSA (Figure 1 in the manuscript) and water (Figure B) reveals the behavior of BSA regarding to AA (Figure C). [file 461365.f1.docx]

**Supplementary Materials**

**Figure A:** UV-vis spectra of AA and BSA solution.

**Figure B:** Spectra of water at pH 7 after addition of AA at different concentrations

**Figure C:** Variation of the absorbance with concentration of AA, obtained from the differences between the spectra of Figure B and Figure 1 (in the manuscript).
